# Neurodevelopmental disorders and the gut microbiome: insights into ADHD and tic disorders

**DOI:** 10.3389/fmicb.2026.1779746

**Published:** 2026-05-07

**Authors:** Jiawen Li, Xinyi Qiu

**Affiliations:** 1 Fujian Medical University Union Hospital, Fuzhou, Fujian, China; 2 Women's and Children's Clinical Medical College, Fujian Medical University, Fuzhou, Fujian, China

**Keywords:** attention deficit hyperactivity disorder, gut microbiome, gut–brain axis, host, tic disorders

## Abstract

This review examines the relationship between tic disorders (TD), attention deficit hyperactivity disorder (ADHD), and the gut microbiota within the framework of the gut–brain axis. We summarize current evidence on the clinical characteristics and neurobiological features of TD and ADHD, and discuss how genetic susceptibility, environmental exposures, and dietary factors may interact with gut microbial composition. We further review studies comparing the gut microbiota of affected individuals and healthy controls, with attention to recurrent taxa-level findings, functional hypotheses, and emerging microbiome-targeted interventions such as probiotics and fecal microbiota transplantation. Importantly, most available human studies remain associative rather than mechanistic, and cross-study comparability is limited by heterogeneity in sequencing approaches, cohort characteristics, medication exposure, and dietary control. Overall, current findings support the gut microbiota as a relevant component of neurodevelopmental disorders such as ADHD and TD, while highlighting the need for larger longitudinal and mechanistic studies to clarify causality and therapeutic potential.

## 1 Introduction

Attention-deficit/hyperactivity disorder (ADHD) and tic disorders (TD) are among the most common neurodevelopmental disorders in children and adolescents (Furman, 2005), ADHD is characterized by impulsivity, inattention, and hyperactivity (Association, 2013) and affects approximately 5%−6% of children (Sayal et al., 2018), with 30%−60% continuing to exhibit symptoms into adulthood (Wender et al., 2001). TD typically begins in childhood, often follows a waxing-and-waning course, and frequently co-occurs with ADHD, thereby increasing functional impairment and management complexity (Percinel et al., 2016; Salehi et al., 2016).

In recent years, the gut–brain axis has emerged as a key framework for understanding how intestinal microorganisms may affect neurodevelopment, behavior and cognitive function (Principi and Esposito, 2016; Sandgren and Brummer, 2018). Conversely, brain activity, stress, and neuroendocrine signaling can also modulate gut microbial composition, establishing a two-way communication system that facilitates dynamic interactions between the central nervous system (CNS) and the digestive system.

The human gastrointestinal tract is home to a complex and varied microbial population that significantly impacts the physiological functions of the host (Gulas et al., 2018; Rinninella et al., 2019). Within this bidirectional system, gut microbial alterations have been associated with neural, immune, endocrine, and metabolic processes that may be relevant to ADHD and TD (Kho and Lal, 2018; Singh et al., 2021; Chilicka et al., 2022; Nowicka et al., 2022). The capacity of gut-derived metabolites to influence brain activity highlights the complex relationship between gut health and mental wellbeing, presenting potential avenues for therapeutic approaches in neurodevelopmental disorders (Dinan and Cryan, 2017; Latalova et al., 2017; Enaud et al., 2018; Daniel, 2020).

Growing evidence suggests that disruptions in the gut–brain communication network could play a role in the development or worsening of neurodevelopmental and neuropsychiatric disorders, such as ADHD and TD (Mitsou et al., 2008; Zhu et al., 2013; Dolina et al., 2014; Shao et al., 2019). However, available evidence remains heterogeneous, and the distinct impact of factors like diet, gastrointestinal comorbidities, and sex differences on microbiota composition are not fully understood (Baikie et al., 2014; Yap et al., 2021; Laue et al., 2022; Tarui et al., 2022).

Emerging studies increasingly highlight the role of gut microbiota imbalances in the onset and progression of neurodevelopmental disorders such as ASD and ADHD. These studies primarily focus on how microbial-associated changes may modulate neurotransmitter-related pathways, precursor availability, and immune regulation. However, the gut–brain axis functions through a complex communication network involving neural, immune, and metabolic pathways. In this context, investigating the gut microbiota's role in neurodevelopmental disorders is crucial, as it could provide valuable insights into their pathophysiology and open new avenues for therapeutic interventions.

Rather than assuming causality, this review aims to examine gut–brain–microbiota axis alterations in ADHD and TD, while considering both shared and disorder-specific mechanisms. We summarize current evidence on gut microbiota alterations in ADHD and TD, with particular attention to mechanistic plausibility, methodological heterogeneity, and microbiome-targeted therapeutic implications. We first outline the clinical characteristics and overlap between ADHD and TD, then discuss the major pathways of gut–brain communication, and finally synthesize current evidence on microbial signatures, pathophysiological interpretations, and intervention studies in these disorders.

## 2 Attention deficit hyperactivity disorder (ADHD) and tic disorder (TD)

### 2.1 Overview of attention deficit hyperactivity disorder

Attention deficit hyperactivity disorder (ADHD) stands as a prevalent neurodevelopmental disorder in the pediatric population (First, 2013). It typically manifests prior to the age of 12 years and is most frequently diagnosed in school-aged children. The symptoms associated with this disorder often persist for a duration of 6 months or longer. ADHD is distinguished by a developmental imbalance when compared to the typical developmental trajectory of children of the same age group. The cardinal clinical features of ADHD encompass hyperactivity, inattention, and impulsivity. These symptoms frequently co-occur with a range of challenges, including academic or occupational difficulties, as well as emotional and behavioral dysregulation (Wilens et al., 2009). Notably, individuals with ADHD generally exhibit an intelligence quotient (IQ) that is average or near-average (Committee on Quality Improvement, Subcommittee on Attention-Deficit/Hyperactivity Disorder, 2000). The etiology of ADHD remains elusive, with current understanding suggesting a multifactorial origin involving a multifaceted interaction of genetic, social, and environmental influences. Various risk factors have been identified for ADHD, including male gender and being the first-born child. Furthermore, ADHD often coexists with tic disorder (TD), and its presence exerts a substantial impact on academic performance and family wellbeing (Canals et al., 2016).

### 2.2 Overview of tic disorders

Tic disorder (TD) is a neurological condition that typically emerges during childhood. It is characterized by the presence of sudden, rapid, and repetitive motor movements or vocalizations that lack a rhythmic pattern (Johnson et al., 2023). These tics may be preceded or accompanied by premonitory sensations or urges. It is worth noting that there appears to be a gender disparity in the prevalence of TD, with males being approximately four times more likely to be affected than females (Pringsheim, 2017). TD often has its initial onset between the ages of 4 and 6 years, and the peak severity of symptoms is commonly observed between 10 and 12 years of age. Symptoms tend to exacerbate during periods of social and psychological stress, emotional distress, and fatigue (Leckman et al., 2014). Current research suggests that TD is a complex genetic disorder, with multiple genes believed to contribute to its pathogenesis. It is characterized by a combination of rare genetic or neonatal alterations and common risk variants. Besides genetic factors, non-genetic elements also contribute significantly to the variability of clinical manifestations seen in TD. Factors such as prenatal events and immune system influences have been linked to the development of both anatomical and functional brain abnormalities, as well as neurocirculatory dysfunction observed in TD patients (Robertson et al., 2015). Fortunately, the prognosis for many children with TD is relatively favorable. By early adulthood, nearly 75% of children with TD experience a substantial reduction in symptoms, and over 33% no longer exhibit any tics (Leckman et al., 2014). However, it is common for patients with TD to have comorbid mental or behavioral disorders, with ADHD being the most prevalent. This co-occurrence significantly impacts the overall prognosis and management of TD, necessitating a comprehensive and interdisciplinary approach to treatment.

### 2.3 Relationship between TD and ADHD

Recent studies have clarified that the coexistence of TD and ADHD is associated with greater clinical complexity compared than either condition alone (Jiang et al., 2025). Children with comorbid TD and ADHD tend to show more severe disruptive behaviors, greater functional impairment, and a less favorable psychosocial prognosis than those with TD alone (Cao et al., 2022). These comorbidities can significantly impair social functioning, negatively impact social behavior and emotional development, pose increased challenges in treatment, reduce the effectiveness of clinical interventions, and result in a less favorable prognosis. In a study conducted by (Martino et al. 2013), no notable differences were found between children with TD alone and typical individuals regarding parental evaluations of aggressive and delinquent behaviors, as well as teacher evaluations of behavioral issues. However, children with comorbid TD and ADHD demonstrated significantly higher scores on these disruptive behavior indicators compared to typical individuals and exhibited comparable levels of disruptive conduct to children with ADHD alone (Xiong et al., 2024). These results highlight the comorbidity is not merely additive at the symptom level, but may reflect partially overlapping yet non-identical neurobiological processes (Greimel et al., 2008). ADHD usually shows a more persistent developmental course characterized by inattention, hyperactivity, and impulsivity, whereas TD is characterized by waxing-and-waning motor or vocal tics and may, in selected subgroups such as PANDAS/PANS, show stronger links to immune dysregulation (Strandwitz, 2018; Cryan et al., 2019). Consequently, microbiota-based approaches—including dietary adjustments, prebiotic and probiotic supplementation, or fecal microbiota transplantation—are being investigated as potential adjunctive therapies rather than disorder-independent solutions (Vendrik et al., 2020; Levy Schwartz et al., 2024).

From microbiome perspective, these clinical overlaps raise the possibility that shared gut–brain pathways may contribute to symptom co-occurrence. At present, however, this hypothesis remains preliminary, and the biological weight of these pathways may not be identical in ADHD and TD; most supporting evidence remains associative rather than causal (Prehn-Kristensen et al., 2018; Ma et al., 2019; Van de Wouw et al., 2018; Sgritta et al., 2019).

The potential contribution of microbiota-related mechanisms is therefore discussed separately in the following section.

## 3 The gut–brain axis

The gut–brain axis acts as a crucial communication link between the gut microbiota and the central nervous system (CNS). This bidirectional pathway allows for the exchange of signals that influence both gut function and brain activity. Current evidence indicates that microbiome–brain interactions occur mainly through neural pathways, immune system modulation, and endocrine pathways, which may contribute to the pathophysiology of neurodevelopmental disorders like ADHD and TD (Martino et al., 2020). Dysbiosis may disrupts microbiota-related metabolic pathways, especially microbiome, epigenome, and metabolome. Thereby affecting the production of neurotransmitters-related process relevant to brain function. In particular, gut microbiota have been implicated in the regulation of dopamine, serotonin, and GABA, while microbial metabolites such as short-chain fatty acids (SCFAs), may affect neuroinflammatory signaling and microglial activity. Moreover, In addition, microbial metabolism of tryptophan and vitamin B6 may further affect neurotransmitter-related pathways (Góralczyk-Bińkowska et al., 2022; Wang et al., 2023). Pyridoxal phosphate (PLP), the active form of vitamin B6, is an essential coenzyme in the metabolism of norepinephrine, tryptophan, serotonin, dopamine, and GABA. Research indicates that reduced PLP-dependent enzyme activity has been reported in children with ADHD (Wang et al., 2025).

The gut–brain communication also occurs through microbial byproducts that directly or indirectly influence gut–brain signaling. Microbial products such as peptidoglycan and SCFAs may affect neurotransmitter signaling and immune responses in the CNS. These microbial products may influence the brain indirectly by modulating serotonin, norepinephrine, dopamine, glutamate, and GABA signaling. For instance, *Bifidobacterium* and *Lactobacillus* synthesize GABA, while *Candida, Escherichia, Enterococcus*, and *Streptococcus* are known as serotonin producers (Socała et al., 2021). These compounds are better interpreted as gut-derived neuroactive signals than as direct central neurotransmitters, because most peripherally produced neurotransmitters do not cross the blood-brain barrier in physiologically relevant amounts. In addition to neurotransmitter synthesis, SCFAs produced by gut microbiota modulate immune responses in the CNS. SCFAs interact with immune cells and intestinal epithelial cells to regulate inflammation, suggesting that microbial metabolites could serve as therapeutic targets for mitigating neuroinflammation and neuropsychiatric disorders (Strandwitz, 2018). Despite the fact that the blood–brain barrier (BBB) restrict the direct passage of many compounds, microbiota-derived signals may still influence the brain through the enteric nervous system (ENS), immune modulation, and microbial regulation of tryptophan metabolism. By influencing the availability of tryptophan and downstream metabolites, gut microbes may affect serotonin-related pathways as well as the production of kynurenine and indole derivatives (Dicks, 2022). These interactions support the view that the gut microbiome may influence emotion, cognition, and motor function, and may therefore be relevant to the neurobiological abnormalities observed in ADHD and TD (Shirvani-Rad et al., 2022).

Earlier studies have elucidated the pivotal role of gut microbiota in immunoregulation. Gut dysbiosis may increase intestinal permeability and inflammation, thereby disrupting immune control and eliciting mild systemic inflammation. In ADHD, such low-grade systemic inflammation may contribute to neuroinflammation and gradual impairment of the BBB (Boonchooduang et al., 2020). SCFAs emerge as key mediators in this communication network because they interact with intestinal epithelial cells and immune cells, promote gut homeostasis, and regulate systemic inflammatory responses (Dalile et al., 2019). They may also mitigate neuroinflammation through immune-related mechanisms (Wang et al., 2023).

**Figure 1:**
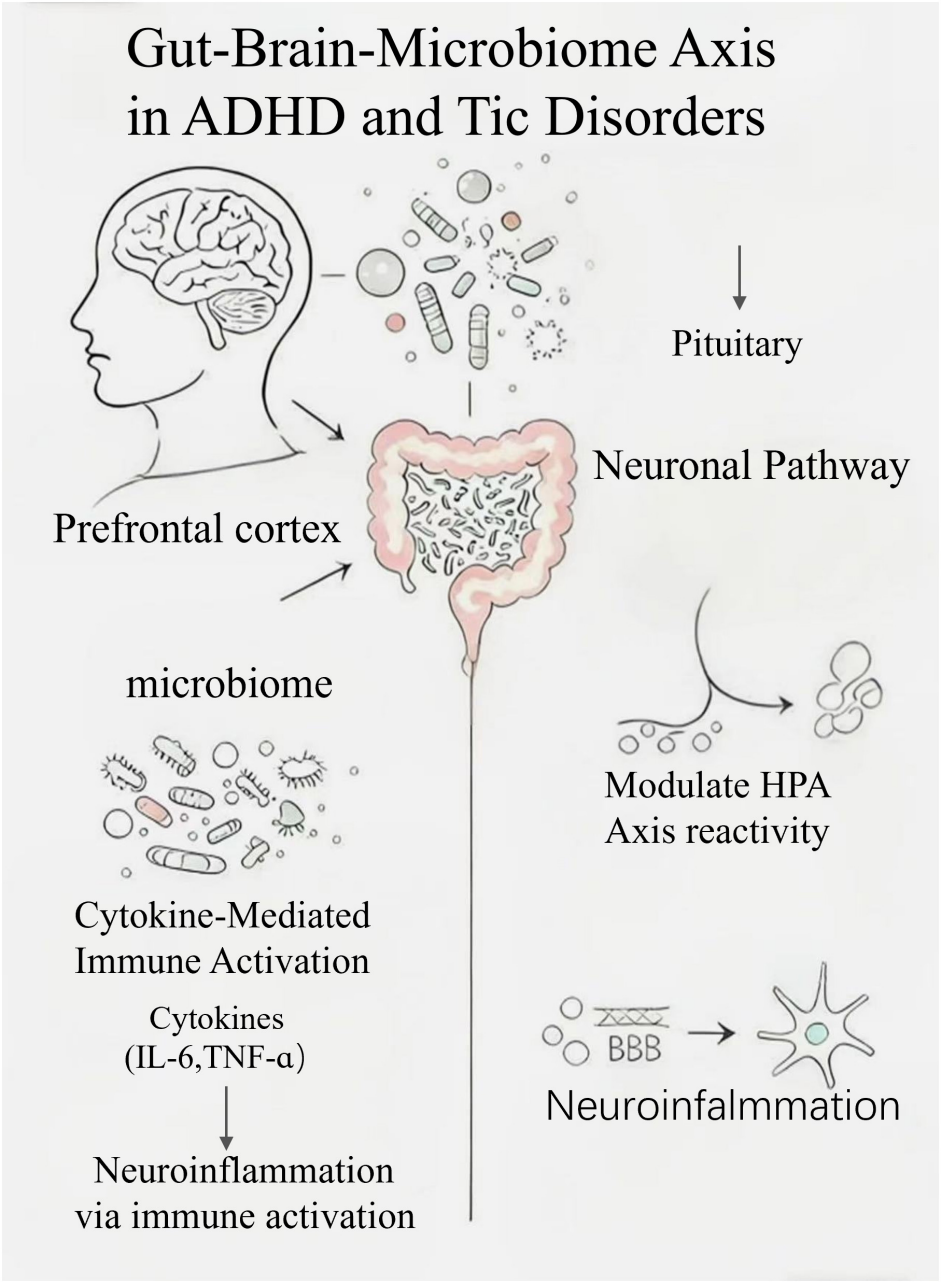
The gut–brain–microbiome axis in ADHD and tic disorders illustrates the intricate two-way communication between the gut microbiota and the brain. This pathway includes both direct microbial activation and immune-mediated mechanisms. The neuroendocrine pathway shows how microbial metabolites can influence the hypothalamic–pituitary–adrenal (HPA) axis, resulting in cortisol release, which regulates stress responses and maintains immune system balance. Additionally, cytokine-mediated immune activation involves key pro-inflammatory cytokines like IL-6 and TNF-α contributing to neuroinflammation. Microbial products also directly activate the brain through interactions with the blood–brain barrier (BBB), affecting microglia and contributing to neuroinflammation. The interplay of these mechanisms can affect regions of the brain such as the prefrontal cortex, which is crucial in the regulation of behavior, emotions, and motor function, and may be implicated in conditions like ADHD and tic disorders.

**Figure 2:**
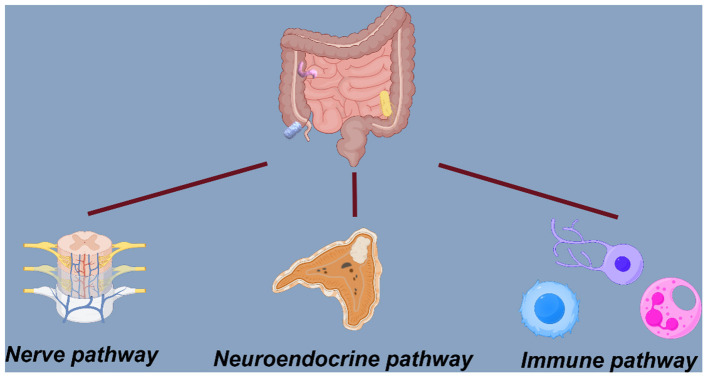
Schematic representation showing the three pathways (neural, neuroendocrine, and immune) that constitute the microbiome–gut–brain axis.

The two-way interaction between the microbiota, enteric nervous system (ENS), and central nervous system (CNS) exemplifies the central role of the gut–brain axis in regulating health and disease (Barrio et al., 2022). Current evidence highlights three established routes of communication: neurological, immunological, and endocrine (Bull-Larsen and Mohajeri, 2019; Kalenik et al., 2021). Stress and pro-inflammatory cytokines trigger the HPA axis, leading to the production of ACTH and CRF, and altered cortisol levels have been correlated with the severity of neurodevelopmental symptoms (Chang et al., 2020). Gut microbes also influence microglial activation, which is relevant to autoimmunity, neuroinflammation, and neurogenesis and dysregulated microglial activity have been observed in germ-free mice (Checa-Ros et al., 2021). In addition, individuals diagnosed with ADHD or TD frequently exhibit autonomic dysregulation, characterized by heightened sympathetic activity and reduced parasympathetic tone, which may contribute to stress reactivity and emotional instability. Together, these findings support the gut–brain axis as a relevant framework for understanding these disorders and a potential target for future therapeutic approaches (Davoli-Ferreira et al., 2021). As shown in Figures 1 and 2, the microbiome-gut-brain axis involves interconnected neural, neuroendocrine, and immune pathways.

## 4 The role of gut microbiome in ADHD

### 4.1 Differences in gut microbiota composition in individuals with ADHD

Representative studies on gut microbiota alterations and interventions in ADHD are summarized in Table 1. The influence of gut microbiota on attention deficit hyperactivity disorder (ADHD) has attracted increasing attention, and current studies suggest that ADHD-related differences may be observed at both the community and taxon levels. However, findings on overall microbial diversity remain inconsistent. Early research by (Aarts et al. 2017) set the stage by exploring the gut microbiota in young adults diagnosed with ADHD. By employing 16S rDNA next-generation sequencing on fecal samples, the study revealed no significant differences in alpha or beta diversity between ADHD patients and healthy controls. In contrast, (Prehn-Kristensen et al. 2018) reported notable alterations in both alpha and beta diversity in the gut microbiota of adolescents with ADHD, with specific microbial taxa linked to the disorder. This finding, coupled with their examination of vertical transmission of microbiota from parents to children, emphasizes the potential for early-life microbial influences on ADHD. In a longitudinal study by (Casas et al. 2019), the researchers observed that lower bacterial diversity at age 10 was inversely associated with the subsequent onset of ADHD, with a greater presence of fungal species correlated with ADHD onset. These results suggest that diversity-related changes may be linked to ADHD, but the direction and magnitude of such associations appear to vary across age groups and developmental stage, sample source, medication exposure, and study design.

**Table 1:** Alterations in gut microbiota following interventions for pediatric ADHD.

References	Design	Sample	Country	Methods	Intervention	Key findings	Limitations
(Aarts et al. 2017)	Cross-sectional case-control + fMRI	19 ADHD, 77 controls; adolescents/young adults (16–25 years)	Netherlands	16S rRNA sequencing (V4); PICRUSt functional prediction; fMRI	None	↑*Bifidobacterium* (nominal); ↑ predicted enzyme for dopamine precursor synthesis; linked to ↓ ventral striatal reward response (*p* < 0.05)	Small ADHD sample (*n* = 19); cross-sectional; 16S not WGS; mixed-age controls
(Prehn-Kristensen et al. 2018)	Case-control	14 ADHD boys (~12 years), 17 male controls (~13 years)	Germany	16S rDNA (V1–V2)	None	↓ Alpha diversity (Shannon p = 0.036); β diversity differences (ANOSIM *p* = 0.033, ADONIS *p* = 0.006); ↑*Bacteroidaceae*; controls: ↑*Prevotellaceae*	Small, all-male sample; most ADHD on methylphenidate; cross-sectional
(Boonchooduang et al. 2020, 2025)	Cross-sectional	10 ADHD (no meds), 10 ADHD (on meds), 10 controls; 6–12 years	Thailand	16S rRNA (V3–V4); fecal SCFAs	Psychostimulant medication (observational)	Unmedicated ADHD: ↓*Tyzzerella, Prevotellaceae*; SCFA propionate inversely related to severity; medicated ADHD:↓ diversity, distinct taxa, lower SCFAs	Very small sample; cross-sectional; medication vs. disease effects unclear
(Pärtty et al. 2015)	RCT, 13-year follow-up	75 infants randomized (40 probiotics, 35 placebo)	Finland	FISH, qPCR (infancy and adolescence)	*Lactobacillus rhamnosus* GG (0–6 months) vs. placebo	At 13 years: ADHD/ASD in 6/35 placebo vs. 0/40 probiotic (*p* = 0.008); infants with later ADHD/ASD had ↓*Bifidobacterium* at 6 months (*p* = 0.03)	~50% attrition by 13 years; not powered for ADHD outcome; observational follow-up
(Cickovski et al. 2023)	Secondary analysis of case-control data	28 ADHD (15 F/13 M), 30 controls (15 F/15 M); 18–25 years	USA	16S rRNA (V3–V4); SparCC network	None	Traditional comparisons largely null; altered microbial network structure in ADHD (*Bacteroides, Faecalibacterium, Streptococcus* clusters)	Small sample; re-analysis; descriptive; no independent replication
(Stiernborg et al. 2023)	Cross-sectional	84 adult ADHD (~33 years), 52 adult controls; 63 child ADHD (33 medicated, 30 unmedicated)	Sweden/USA	Shotgun metagenomics; plasma cytokines/SCFAs	Psychostimulant medication (child subgroup)	Adults: taxonomic and functional β diversity differences (*p* < 0.05); plasma inflammatory markers	Cross-sectional; medication confounders; modest control numbers
(Cassidy-Bushrow et al. 2023)	Prospective birth cohort (WHEALS)	314 infants; 59 ADHD diagnoses by age 10	USA	16S rRNA + ITS2 (1 and 6 months)	None	6 months: microbiota differed between ADHD and non-ADHD (UniFrac *p* = 0.006, PD *p* = 0.017); 1 month: 18 bacterial and three fungal OTUs linked to later ADHD; 6 months: 51 bacterial OTUs (14 *Lactobacillales*) and three fungal OTUs linked to later ADHD	Observational; ADHD based on parental report; urban cohort; only 16S/ITS
(Wan et al. 2020)	Case-control	Children with ADHD vs. healthy controls	China	16S rRNA sequencing	None	ADHD: ↓*Faecalibacterium*, ↓*Veillonellaceae*; ↑*Odoribacter*, ↑*Enterococcus*	Small sample; cross-sectional; dietary/environmental confounders
(Stevens et al. 2019)	10-week intervention trial	17 ADHD children (seven placebo, 10 micronutrient)	New Zealand	16S rRNA sequencing	Broad-spectrum micronutrient supplementation vs. placebo	↑ OTU richness; ↓ Actinobacteria (mainly *Bifidobacterium*); compensatory ↑*Collinsella*	Very small sample; short-term; limited explanatory power
(Han et al. 2025)	Multi-omics case-control study	15 treatment-naïve ADHD vs 15 controls	Multi-country	16S rRNA + plasma metabolome + lipidome	None	↓ B*ifidobacterium*; ↑*Veillonella*; integrative analysis suggests *Bifidobacterium*-mediated neurotransmitter precursor pathways in ADHD	Small single-center cohort; causality untested

At the taxonomic level, a noteworthy finding was the increased abundance of *Bifidobacterium* within the Actinobacteria phylum in ADHD individuals. (Jiang et al. 2018) found a negative correlation between the abundance of *Faecalibacterium* and ADHD symptom severity in untreated children. Similarly, (Wan et al. 2020) found lower levels of *Faecalibacterium* and higher levels of *Odoribacter* and *Enterococcus* in children with ADHD. Collectively, these findings indicate statistically significant differences in selected genera rather than a uniform microbial signature across all studies, and some discrepancies may reflect differences in age, medication status, and analytical platform.

Some authors have further proposed functional interpretations for these taxonomic findings. *Bifidobacterium* was linked to the enzyme cyclohexadienyl dehydratase (CDT), involved in phenylalanine biosynthesis, a precursor to dopamine. This observation raises the possibility that microbial alterations may influence dopamine precursor availability; however, this inference was based on predicted functional potential rather than direct experimental demonstration of causality. Likewise, the reported association between lower *Faecalibacterium* abundance and greater symptom severity (Jiang et al., 2018) may be relevant to immune regulation, but it should also be interpreted as a statistical association rather than proof of mechanism.

Lastly, a study on micronutrient supplementation uncovered a complex connection between gut microbiota and ADHD symptoms. Although no significant changes in microbiome composition were observed, a reduction in *Bifidobacterium* levels was linked to reduced ADHD symptom scores, indicating a possible role for *Bifidobacterium* in managing ADHD symptoms. However, the variability in findings across studies indicates the need for additional studies are needed to elucidate the role of specific microbiota in ADHD. Taken together, current evidence supports an association between gut microbial alterations and ADHD, but heterogeneity in sequencing methods, sample size, medication exposure, and dietary confounding limits firm mechanistic conclusions. Therefore, taxa such as *Bifidobacterium, Faecalibacterium*, and *Ruminococcaceae* should presently be regarded as candidate correlates rather than definitive biomarkers or causal drivers of ADHD.

### 4.2 Gut microbiome is associated with ADHD symptoms and pathophysiological implications

Several studies have reported associations between *Enterococcus* and the release of neurotransmitters, which may result in an overproduction of dopamine from levodopa in the intestines. However, peripheral dopamine agonists cannot cross the blood–brain barrier (BBB) in physiologically meaningful amounts (Maini Rekdal et al., 2019). Additionally, animals lacking the 5-HT transporter have been found to exhibit significantly higher levels of *Enterococcus*. These findings provide mechanistic clues regarding dopaminergic and serotonergic pathways, but they do not directly demonstrate that *Enterococcus* causally drives ADHD symptoms in humans. The elevated levels of *Odoribacter* observed by Wan et al., in ADHD patients are consistent with a prior study that reported a higher prevalence of this genus in pediatric autoimmune neuropsychiatric disorders associated with streptococcal infections (PANDAS) and pediatric acute-onset neuropsychiatric syndrome (PANS) (Quagliariello et al., 2018). This comparison is hypothesis-generating and may point to overlapping inflammatory or neuroimmune features, but it should not be interpreted as direct mechanistic proof in ADHD. The main limitations of this study were the lack of information on the usage of ADHD medication and the small sample size. Moreover, the investigators did not find any correlations between the fundamental symptoms of ADHD and the microbial composition. The study's quality is bolstered by its rigorous methodology, which includes a comprehensive description of the recruitment process, the use of whole genome shotgun sequencing (WGS), and the evaluation of confounding factors in both groups, including probiotics, allergic disorders, respiratory or digestive problems, and dietary habits. Whole genome sequencing (WGS) offers a more reliable assessment of the microbiome's functional potential compared to 16S rDNA subunit sequencing (Cryan et al., 2019).

(Casas et al. 2019) conducted a case-cohort study to explore the impact of early-life indoor microbial diversity and later hyperactivity/inattention. In this birth-cohort-based analysis, early indoor microbial exposure was characterized and related to behavioral outcomes during follow-up. Bacterial taxon richness was negatively correlated with ADHD frequency at age 10. Conversely, there was a positive association between the number of fungal species and a higher incidence of ADHD (Jakobsson et al., 2010). These findings support an association between early-life microbial exposure and later behavioral symptoms, but they do not by themselves establish a causal mechanism.

### 4.3 The gut microbiota's therapeutic function in ADHD

Probiotics are viable microorganisms that confer health benefits to the host when administered in adequate quantities (Camilleri, 2021). Gut microorganisms may influence host physiology through effects on barrier function, metabolite production, and immune regulation, thereby providing biological plausibility for microbiota-targeted interventions. Prebiotics are specific dietary components that are selectively fermented by specific bacteria in the gastrointestinal tract, leading to beneficial effects on the host (Yadav et al., 2022). Accordingly, probiotics and prebiotics have been explored as potential adjunctive strategies in disorders along the gut–brain axis. A study on mice demonstrated that the prebiotic Bimuno^®^ galactooligosaccharide (BGOS) enhanced the activity of cortical *N*-methyl-d-aspartate (NMDA) receptors and improved cognitive flexibility in a task related to attentional set-shifting (Tan et al., 2021). However, this finding constitutes preclinical evidence and should not be taken as direct clinical proof in ADHD.

Human evidence is encouraging but still limited, and most studies are better interpreted as early developmental or adjunctive investigations than as definitive therapeutic trials. In an intervention trial involving 75 neonates from Finland, the impact of *Lactobacillus rhamnosus GG* supplementation during the first 6 months of life on the gut microbiota of children at risk for ADHD was investigated in a comprehensive 13-year randomized controlled trial (RCT). FISH and qPCR were employed to analyze the fecal microbiota at six different time points, ranging from 3 weeks to 2 years of age (Kumperscak et al., 2020). The fecal microbiota was reassessed at the age of 13, coinciding with the diagnosis of ADHD. Among the six individuals who received a placebo, Asperger's syndrome or ADHD was diagnosed, whereas none of the patients who received *Lactobacillus rhamnosus GG* supplementation were diagnosed with these conditions. The study focused on examining specific bacteria using FISH and qPCR methodologies at designated time points. A decrease in the number of *Bifidobacterium longum* was observed at 3 months, along with a decline in the overall *Bifidobacterium* genus at 6 months (Mathee et al., 2020). At 18 and 24 months, *Lactobacillus–Enterococcus* and *Clostridium histolyticum* were found to be reduced compared to age-matched controls. However, when comparing the fecal microbiota composition and structure of 13-year-olds with ADHD or Asperger's syndrome (AS) to their control group, no discernible alterations were found. These findings suggest a possible preventive effect of early probiotic supplementation, but the evidence remains limited and does not conclusively establish causality. Nonetheless, the study was limited by a small sample size and outdated technology that was unable to distinguish specific species of gut microbiota linked to the disease (Allahyari et al., 2024).

Beyond early-life intervention, broader microbiota-targeted strategies have also been considered, but the strength of evidence remains uneven. Proposed benefits are biologically plausible, yet most mechanistic links are inferred rather than experimentally verified in ADHD patients, and no microbiota-targeted approach can yet be regarded as an established treatment for core ADHD symptoms. Probiotics, which are living microorganisms that improve the host's physiological state when administered in sufficient quantities, may serve as dietary components, supplements, or pharmaceuticals to provide health advantages. Currently, strains of the genera *Lactobacillus* and *Bifidobacterium* have been extensively studied (Liu et al., 2023). A thorough review examined randomized controlled studies that evaluated the effects of probiotic supplementation on cognitive performance in children and adolescents conducted between 1990 and 2018. Of the seven trials examined, only one demonstrated a substantial decrease in the likelihood of developing ADHD or ASD. As discussed above, the long-term LGG study by (Pärtty et al. 2015) provides the strongest human signal, but it should be interpreted cautiously because of its modest sample size, attrition, and limited microbial resolution. Nevertheless, it is important to consider some constraints when analyzing these findings: a significant proportion of participants who discontinued the study (approximately 50%) may have influenced the results. The study did not collect any data on the dietary habits of mothers during pregnancy and after delivery for breastfed infants. Furthermore, the research did not provide any information on possible risks associated with ADHD, such as maternal smoking, dietary inadequacies, and psychiatric issues. The presence of *Bifidobacterium* at 6 months of age did not correlate with psychometric scores for ADHD (Han et al., 2025).

In summary, the gut microbiota has been recognized as a potential target for intervention in various neuropsychiatric diseases, including ADHD. However, the constraints of these studies, such as the absence of comprehensive microbial composition analysis, necessitate further research to validate its potential advantages (Wang et al., 2022a).

## 5 The function of the gut microbiome in TD

### 5.1 Differential gut microbial profiles in patients with TD

Representative human and animal studies on gut microbiota alterations and microbiome-targeted interventions in TD/TS are summarized in Table 2. Tic disorders (TD) present as multifactorial neurodevelopmental conditions, influenced by genetics, environment, and social factors, though their full origins are still debated. Emerging studies have suggested a possible association between gut microbiota imbalance and TD, but the available evidence remains preliminary and methodologically heterogeneous, with differences in study populations, sequencing platforms, and control of confounding factors making direct comparison difficult (Wan et al., 2020). Compared with the ADHD literature, the TD evidence base is earlier-stage and more fragmented. TD also differs clinically from ADHD in that tic severity often waxes and wanes, and selected subgroups such as PANDAS/PANS suggest a stronger immune-related component.

**Table 2:** Alterations in gut microbiota associated with Tourette's/tic disorders (TD/TS): human and animal studies.

References	Design	Sample	Country	Methods	Intervention	Key Findings	Limitations
(1) (Xi et al. 2021)	Case-control	49 TD children (mean 8.6 years), 50 controls	China	Shotgun metagenomics	None	TD: ↑*Bacteroides_plebeius*, ↑*Ruminococcus_lactaris*; ↓*Prevotella_stercorea, Streptococcus_lutetiensis*; ↑ GABA degradation pathway; classifier AUC = 0.884	Cross-sectional; some on dopamine antagonists; modest sample
(2) (Wang Y. et al. 2022)	Case-control	28 TD children, 21 controls	China	16S rRNA sequencing	None	TD showed compositional differences vs. controls; taxa shifts included ↑*Odoribacter* and changes in *Prevotella*-related groups	Small sample; cross-sectional; dietary factors not fully controlled
(3) (Zhao et al. 2020)	Open-label clinical FMT (pilot)	5 TS patients	China	16S rRNA; clinical YGTSS	FMT (donor stool via endoscopy)	Improved YGTSS; restoration of *Bacteroides_coprocola* correlated with symptom improvement	Open-label; very small sample; no control; short follow-up
(4) (Li et al. 2022)	Animal FMT study (mouse)	TS-model mice (*n* = 80) vs. controls (*n* = 80)	China	16S rRNA; behavior; serum 5-HT	FMT (healthy vs. TS donor)	Healthy-donor FMT: ↓ tic severity; ↑ serum 5-HT; ↑*Turicibacteraceae* and *Ruminococcaceae*	Animal model; 16S limits resolution; translational relevance uncertain
(5) (Wu et al. 2021)	Randomized, double-blind, placebo-controlled	PS128 vs. placebo in children with TS	Taiwan	Clinical RCT; symptom scales	Probiotic *Lactobacillus* plantarum PS128	Notic reduction vs. placebo; improvements in some comorbid symptoms reported	No microbiome sequencing; sample size modest; short duration
(6) (Bao et al. 2023)	Open-label intervention	32 TS children (3–14 years), 29 controls	China	16S rRNA (V3–V4)	Cranial electrotherapy + biofeedback	Post-treatment: ↓*Dorea, Agathobacter, Bifidobacterium*; predicted ↓ dopamine degradation modules; ↓ YGTSS	Nosham-control; combined interventions; short follow-up
(7) (Zhao et al. 2017)	Case report (FMT)	Single TS child	China	Clinical observation	FMT	Marked symptom amelioration after FMT	Single case; no microbiome profiling; uncontrolled
(8) (Liang et al. 2025)	Clinical comparative (probiotic vs. clonidine)	Chronic TDs; pediatric cohort	China	Clinical outcomes	*Limosilactobacillus* reuteri vs. *clonidine*	Probiotic showed comparable improvement to clonidine on TD symptoms	Microbiome sequencing; potential biases; not double-blind

At a broad community level, the currently available studies have reported diversity-related changes, but these findings are not yet consistent. (Quagliariello et al. 2018) observed reduced alpha diversity in younger children with PANS/PANDAS, whereas other pediatric TD studies have focused more on compositional shifts than on a reproducible diversity signature (Xi et al., 2021). In this context, the diversity findings should be interpreted as associative observations rather than as evidence of a disease-specific microbial pattern.

Although these studies suggest a potential role of gut microbiota in TD, the research is still in its infancy. The use of small sample sizes, as well as *in silico* predictions of microbial functions rather than direct metabolic measurements, calls for larger, more robust studies to validate these preliminary findings. Furthermore, direct interventions like FMT and probiotic treatments could be explored in future clinical trials to assess their effects on tic severity and neuropsychiatric outcomes.

Overall, the TD literature suggests that gut microbial alterations may be associated with the disorder, but the evidence remains early, scattered, and sensitive to methodological differences. At present, no single microbial profile can be regarded as a robust biomarker for TD, and larger studies with better control of medication, diet, and analytical platform effects are still needed.

### 5.2 The gut microbiome is linked to TD symptoms and pathophysiological consequences

New evidence indicates that the gut microbiome may contribute to clinical features and pathophysiological processes relevant to Tourette's disorder (TD). This neurodevelopmental condition, characterized by motor and vocal tics, has been linked to disruptions in brain areas such as the striatum and prefrontal cortex, which are involved in tic manifestation. In one study, (Liao et al. 2019) investigated the effects of *Lactobacillus plantarum PS128* in a rat model of TD. They found that administration of this probiotic strain led to a significant reduction in tic-like behaviors, along with enhanced dopamine metabolism and elevated norepinephrine levels in brain regions critical to TD pathophysiology. These findings suggest that gut microbiota modulation may influence neurotransmitter-related pathways implicated in TD, but they should be interpreted as preclinical evidence rather than direct proof of clinical efficacy. However, the study's small sample size and the limitations of the experimental techniques warrant caution, highlighting the need for further validation in more rigorous studies.

At the same time, clinical studies have started to investigate the therapeutic potential of microbiota modulation in human TD patients. A pilot investigation by (Ding et al. 2019) reported a transient reduction in tic severity following the administration of a mixed bacterial population through three infusions in 11 male patients with TD. While these results are promising, the brief duration of the observed effects and the small cohort size highlight the challenges in establishing robust, long-term benefits. Moreover, the methodological limitations, including the lack of detailed mechanistic insights, further underscore the need for larger, more comprehensive trials.

### 5.3 Microbiome-targeted therapeutic interventions

#### 5.3.1 Probiotics and experimental models

Preclinical research has suggested that probiotics can affect the gut–brain axis and ameliorate tic-like behaviors, but these findings should be interpreted as experimental evidence in animal models rather than direct confirmation of a human therapeutic mechanism. (Liu Z. S. et al., 2020; Cassidy-Bushrow et al., 2023) treated TD-model rats with *Lactobacillus plantarum* PS128 (PS128), a probiotic strain known for its anxiolytic and neuroprotective effects. The TD phenotype was induced using 2,5-dimethoxy-4-iodoamphetamine (DOI), and PS128 modified muscle contraction patterns and microbial composition, increased the relative abundance of *Bacteroidaceae* and *Prevotellaceae*, reduced *Lachnospiraceae*, and was accompanied by more stable dopamine metabolism and higher norepinephrine concentrations in brain regions implicated in TD (Han et al., 2025). These results provide mechanistic clues, but they remain model-dependent. However, inter-study variability and the use of different TD-inducing models (e.g., DOI vs. IDPN) complicate direct comparisons, and animal studies cannot fully capture the microbiota-neurobehavioral interactions observed in children with TD (Maini Rekdal et al., 2019). Translating these findings into clinical contexts, (Wang Y. et al. 2022) carried out a 12-week randomized controlled trial (RCT) with 130 children diagnosed with TD. Participants were randomly assigned to receive either tiapride alone, probiotics (a mixture of *Bifidobacterium, Lactobacillus*, and *Enterococcus*), or combined therapy. The combined treatment group exhibited the most significant reduction in Yale Global Tic Severity Scale (YGTSS) scores, while probiotics alone yielded only modest improvements. These results indicate that probiotics may be more suitable as adjunctive rather than stand-alone, therapies for TD. However, the interpretation of clinical benefit remains affected by strain specificity, treatment duration, dietary variation, and the fact that microbiome sequencing was not performed in all clinical trials (Liang et al., 2025). Standardized formulations and longer follow-up are still needed.

#### 5.3.2 Fecal microbiota transplantation (FMT)

Fecal microbiota transplantation (FMT) has emerged as an experimental microbiome-targeted approach for neuropsychiatric conditions associated with gut microbiota imbalances, including tic disorders (TD) (Vendrik et al., 2020). One case reported by (Zhao et al. 2020) highlighted substantial symptom improvement, with a 9-year-old patient showing a marked reduction in tic severity and enhanced attention (Zhao et al., 2017), and small follow-up studies have also reported symptomatic improvement in some additional TD patients (Zhao et al., 2020). Mouse model studies by (Li et al. 2023), found alleviated tic-like behaviors and elevated serotonin levels following FMT, which provides preclinical support for a microbiota-neurotransmitter connection. Nevertheless, the human evidence is still limited to case reports, open-label studies, and short-term observations (Zhao et al., 2020), so FMT should be presented as a pilot or experimental strategy rather than a well-established therapy. Taken together, microbiome-targeted interventions in TD are promising, but the clinical evidence remains insufficiently consistent and mechanistically unresolved.

## 6 Current limitations and outlook

In the last 5 years, extensive research has investigated the connection between gut microbiota and neurodevelopmental disorders such as ADHD and TD. Although these disorders partly overlap, they should not be treated as microbiologically interchangeable conditions. In children with ADHD or TD, there is often a reduction in gastrointestinal symptoms such as constipation, abdominal distension, and stomach pain. However, these symptoms may be underreported due to the challenges children face in articulating them. At the same time, changes in the gut microbiota can more directly impact the nervous system, emotional regulation, and behavior, including irritability, hyperactivity, and autism spectrum traits, mainly through indirect metabolic, immune, endocrine, and neural pathways. Alterations in the gut microbiota, including an increase in *Ruminococcaceae* and *Bacteroides* species (Borrego-Ruiz and Borrego, 2024), such as *Ruminococcus lactaris* and *Bacteroides plebeius*, have been observed in individuals with these disorders (Liu et al., 2025). Despite these findings, using gut microbiota profiling as a diagnostic tool for ADHD or TD remains inconclusive. Studies, such as Li's attempt to transfer fecal microbiota from TD mice to healthy mice without inducing tic-like behaviors, highlight the complexity of establishing a causal relationship between microbial changes and disorder onset (Li et al., 2022). Further animal studies are required to clarify the role of the gut microbiota in the development and progression of these neurological disorders.

Numerous factors—genetic predisposition, medication, diet, cultural background, and environmental influences—affect gut microbiota composition. Given the intricate interaction between gut bacteria and the nervous system, comprehensive research is essential. Additionally, the mechanisms through which interventions like probiotics and fecal microbiota transplantation (FMT) influence these conditions remain unclear, underscoring the need for further investigation (Charitos et al., 2024).

In clinical settings, increasing attention is being given to microbiota-based treatments for TD and ADHD, including FMT and probiotics. Traditional therapies such as acupuncture, massage, and herbal medicine have also demonstrated efficacy in managing TD symptoms by modulating the gut microbiota. Notably, a study by (Wang et al. 2022b) showed that acupuncture and massage alleviated symptoms in 35 out of 40 children with TD, with their gut microbiota subsequently resembling that of healthy individuals. Additionally, Qinglong Zhidong Decoction, a traditional Chinese medicine, has been shown to modify gut flora, enhancing beneficial bacteria like *Lactobacillus* and *Bacteroides* while reducing harmful bacteria like *Alloprevotella* and *Akkermansia*. While these therapies show promise with minimal side effects compared to antipsychotic treatments, their clinical application remains limited due to small sample sizes and the lack of large-scale studies. Thus, further clinical trials and animal experiments are necessary to evaluate their effectiveness and safety.

Another major limitation is the absence of predictive microbiome biomarkers for ADHD and TD. Future work should integrate taxonomic, metabolomic, immune, and clinical data using standardized protocols, and should compare shared vs. disorder-specific pathways rather than assuming a single microbiome signature across ADHD and TD, so that microbial signals can be interpreted in a more biologically meaningful and clinically translatable way. In the future, large-scale, multicenter studies will be crucial for determining the functional significance of gut microbiota in these disorders and for developing new, more effective treatments. This research will also contribute to the creation of standardized methodologies and comprehensive databases for microbiome studies, advancing our understanding of gut–brain interactions.
